# Hemodynamic Status During Endovascular Stroke Treatment: Association of Blood Pressure with Functional Outcome

**DOI:** 10.1007/s12028-021-01229-w

**Published:** 2021-06-17

**Authors:** Min Chen, Dorothea Kronsteiner, Johannes Pfaff, Simon Schieber, Laura Jäger, Martin Bendszus, Meinhard Kieser, Markus A. Möhlenbruch, Peter A. Ringleb, Julian Bösel, Silvia Schönenberger

**Affiliations:** 1 Department of Neurology, Heidelberg University Hospital, Heidelberg, Germany; 2 Institute of Medical Biometry and Informatics, Heidelberg University, Heidelberg, Germany; 3 Department of Neuroradiology, Heidelberg University Hospital, Heidelberg, Germany; 4 Department of Neurology, Kassel General Hospital, Kassel, Germany

**Keywords:** Acute ischemic stroke, Thrombectomy, Blood pressure

## Abstract

**Background:**

Optimal blood pressure (BP) management during endovascular stroke treatment in patients with large-vessel occlusion is not well established. We aimed to investigate associations of BP during different phases of endovascular therapy with reperfusion and functional outcome.

**Methods:**

We performed a post hoc analysis of a single-center prospective study that evaluated a new simplified procedural sedation standard during endovascular therapy (Keep Evaluating Protocol Simplification in Managing Periinterventional Light Sedation for Endovascular Stroke Treatment). BP during endovascular therapy in patients was managed according to protocol. Data from four different phases (baseline, pre-recanalization, post recanalization, and post intervention) were obtained, and mean BP values, as well as changes in BP between different phases and reductions in systolic BP (SBP) and mean arterial pressure (MAP) from baseline to pre-recanalization, were used as exposure variables. The main outcome was a modified Rankin Scale score of 0–2 three months after admission. Secondary outcomes were successful reperfusion and change in the National Institutes of Health Stroke Scale score after 24 h. Multivariable linear and logistic regression models were used for statistical analysis.

**Results:**

Functional outcomes were analyzed in 139 patients with successful reperfusion (defined as thrombolysis in cerebral infarction grade 2b–3). The mean (standard deviation) age was 76 (10.9) years, the mean (standard deviation) National Institutes of Health Stroke Scale score was 14.3 (7.5), and 70 (43.5%) patients had a left-sided vessel occlusion. Favorable functional outcome (modified Rankin Scale score 0–2) was less likely with every 10-mm Hg increase in baseline (odds ratio [OR] 0.76, *P* = 0.04) and pre-recanalization (OR 0.65, *P* = 0.011) SBP. This was also found for baseline (OR 0.76, *P* = 0.05) and pre-recanalization MAP (OR 0.66, *P* = 0.03). The maximum Youden index in a receiver operating characteristics analysis revealed an SBP of 163 mm Hg and MAP of 117 mm Hg as discriminatory thresholds during the pre-recanalization phase to predict functional outcome.

**Conclusions:**

In our protocol-based setting, intraprocedural pre-recanalization BP reductions during endovascular therapy were not associated with functional outcome. However, higher intraprocedural pre-recanalization SBP and MAP were associated with worse functional outcome. Prospective randomized controlled studies are needed to determine whether BP is a feasible treatment target for the modification of outcomes.

**Supplementary Information:**

The online version contains supplementary material available at 10.1007/s12028-021-01229-w.

## Introduction

Today, the periinterventional, or at least the postinterventional, care of endovascular stroke treatment (EST) is increasingly managed by specialized physicians with neuroanesthetic expertise. Optimal blood pressure (BP) management during acute EST is not well established. Current guidelines recommend maintaining the systolic BP (SBP) under 180–185 mm Hg and above 140 mm Hg, as well as avoiding excessive BP drops during thrombectomy, with low to moderate levels of evidence [1–4].

Extreme levels of BP during an acute ischemic stroke may be detrimental, with a U-shaped relation between BP and functional outcome [5, 6]. During the acute phase of ischemic stroke, BP is frequently elevated, which might constitute a necessary compensatory mechanism for maintaining cerebral perfusion [7, 8]. On the other hand, maladaptively high systemic BP might lead to cerebral edema and hemorrhage [5].

EST has now become the recommended treatment for acute ischemic stroke due to large-vessel occlusion [9]. During EST, sedating medications are often used during the intervention, e.g., because of agitation, and their hemodynamic effect has to be monitored and, possibly, treated.

In this post hoc analysis of the Keep Evaluating Protocol Simplification in Managing Periinterventional Light Sedation for Endovascular Stroke Treatment (KEEP SIMPLEST) study [10], we aimed to identify potential associations of BP variations during four different phases of EST with short-term and long-term functional outcome and reperfusion in the setting of procedural sedation and with protocol-based BP management.

## Methods

### Design of the KEEP SIMPLEST Study

Keep Evaluating Protocol Simplification in Managing Periinterventional Light Sedation for Endovascular Stroke Treatment [10] was a prospective observational single-center study comparing the outcome of patients undergoing EST in procedural sedation under an optimized, lean standard operating procedure (SOP), with the preceding SOP in the procedural sedation arm of the Sedation vs. Intubation for Endovascular Stroke Treatment (SIESTA) trial [11]. To compare both SOPs, a propensity score matching approach was applied to the study cohort and the historical SIESTA cohort. Briefly, the new standard comprised managing patients preferentially in procedural sedation. When sedation was deemed necessary (by the interventionalist and/or neuroanesthetist), propofol and esketamine were initiated as short-acting sedatives and administered fractionized and/or continuously. Esketamine was used as a sedative to reduce the need for catecholamines and opioids. If patients still showed signs of pain, remifentanil for analgesia was applied per infusion. Applied doses are shown in Supplementary Table 1. A small proportion of patients (8.8%) did not need sedative medication intraprocedurally. Endotracheal intubation was performed when patients remained agitated or showed symptoms of respiratory insufficiency or when airway patency was lost. Furthermore, different simplified operational optimizations, such as better and faster communication sequences and the improvement of equipment by introducing a new mobile EST cart, were implemented in the new SOP. In our center, periinterventional care is performed by neurologists trained in monitored neuroanesthetic care.

The BP protocol specified an SBP target between 140 and 160 mm Hg and an avoidance of values less than 120 or more than 180 mm Hg during the pre-recanalization and post-recanalization phases. This target range has been chosen because retrospective studies suggested that an SBP less than 140 mm Hg is associated with a worse functional outcome [12, 13]; on the other hand, very high SBP [14] levels are also associated with a poor outcome.

The sample size was calculated after an interim analysis and led to the inclusion of 161 patients, of whom 69 were paired with the historic cohort of the SIESTA trial via propensity score matching.

Main clinical and procedural findings of KEEP SIMPLEST showed that early neurological improvement and modified Rankin Scale (mRS) scores at 3 months were not different compared with the preceding approach in the procedural sedation group of SIESTA, but operational advantages, such as reduced in-house treatment times, were achieved [10].

The study was approved by the local institutional review board (Ethikkommission Medizinische Fakultät Heidelberg, ID S-325/2015). There was no external funding for this study.

### Data Collection

Data from 161 patients were collected in the KEEP SIMPLEST study from December 2016 to November 2017. To investigate possible associations between BP values and reductions from baseline and different outcome parameters, we decided to only analyze the subset of 139 patients with successful recanalization (defined as thrombolysis in cerebral infarction [TICI] grade 2b–3) for early neurological improvement (i.e., change in National Institutes of Health Stroke Scale [NIHSS] score after 24 h) and an mRS score at 3 months because reperfusion status has a large influence on clinical outcomes and we chose to avoid its confounding effects [15]. To evaluate whether BP during the baseline and pre-recanalization phases has an influence on the reperfusion status, mean SBP, mean diastolic BP (DBP), and average mean arterial pressure (MAP) data were analyzed in the total study population. BP was measured noninvasively.

The Alberta stroke program early computed tomography score (ASPECTS) was routinely assessed preinterventionally in non-contrast-enhanced computed tomograpy imaging. ASPECTS and reperfusion status were collected via a chart review. The mRS score at 3 months was obtained via a telephone interview, and NIHSS scores at admission and 24 h after admission were obtained via a chart review.

Different hemodynamic and physiologic parameters, such as SBP, DBP, heart rate, peripheral oxygen saturation, and use of analgesics, sedatives, or catecholamines and their respective doses, were recorded. MAP values were calculated as MAP = (SBP + 2 × DBP)/3. All three BP dimensions (SBP, DBP, and MAP) were used for analysis. We defined four different periinterventional time segments: baseline, pre-recanalization, post recanalization, and post intervention. The segment after groin puncture and before the final recanalization result was specified as the pre-recanalization phase, and afterward as the post-recanalization phase. The mean of up to three BP measurements obtained in the emergency department was specified as the baseline value (baseline BP), and if that value was not recorded, then BP values during the ambulance transport to the emergency department or shortly before the groin puncture were used. After groin puncture, BP was measured noninvasively every 5 min during the EST. We obtained the postinterventional BP values (postintervention BP) from the first three documented noninvasive or invasive BP measurements at the stroke unit or the intensive care unit.

Additionally, we chose to investigate whether a reduction in SBP or MAP of at least 20%, 30%, or 40% from baseline to pre-recanalization was associated with unfavorable functional outcomes at 3 months. A reduction was defined when at least one of the 5-min interval measurements showed a value that was at least 20%, 30%, or 40% lower than the mean baseline BP value.

Doses of bolus injections and perfusion rates for propofol and esketamine, as well as perfusion rates of remifentanil and norepinephrine, were documented for every 5-min interval.

### Exposures

The exposure variables were mean SBP, mean DBP, and mean MAP; changes in mean SBP, mean DBP, and mean MAP between the different phases of EST (from baseline to pre-recanalization, baseline to post recanalization, and baseline to post intervention); and reductions (of more than 20%, 30%, and 40%) in SBP and MAP compared with baseline values.

### Outcomes

The primary outcome was the mRS score after 3 months. Secondary outcomes were change in NIHSS score after 24 h and reperfusion status.

### Statistical analysis

Descriptive statistics were calculated for baseline and follow-up data, including mean and standard deviation (SD) for continuous data, median and interquartile range for scores, and absolute and relative frequencies for categorical data. The BP values were evaluated by the within-individual mean and SD.

The analysis aimed to evaluate the influence of changes between baseline BP and BP in the following phases, as well as mean BP during each phase, on the outcomes. We used linear and logistic regression models for recanalization status (TICI grade 0–2a vs. 2b–3), change in NIHSS score at 24 h, and mRS score at 3 months (0–2 vs. 3–6). Either mean SBP, mean DBP and mean MAP or their respective changes between different phases were included as predictors. Furthermore, the heart rate, baseline NIHSS score, preinterventional ASPECTS, intubation, and medication (propofol, norepinephrine, and esketamine) were included as predictor variables. In models for BP differences between different phases and mRS score, we omitted the medication because the adjustment led to unstable models. In addition, the association between BP drops of 20%, 30%, and 40% and the functional outcomes (change in NIHSS and mRS scores) was evaluated using linear and logistic regression models. Adjusted regression coefficients for linear regression models, or odds ratios (OR) for logistic regression models, with the corresponding confidence intervals (CI) and *P* values are reported. Multiple imputation using chained equations generating 50 data sets and Rubin’s rules are used to handle missing values [16]. To identify thresholds in SBP and MAP to predict good functional outcome (mRS score at 3 months 0–2 vs. 3–6), a receiver operating characteristics (ROC) analysis was used. The thresholds were identified by maximizing the Youden index. The ROC curves, including the area under the ROC curve, and the corresponding sensitivity, specificity, and thresholds are reported.

Because this is an exploratory study, all *P* values are of a descriptive nature, and no adjustment for multiple testing was applied.

The software R (version 4.0.2) was used for statistical analysis [17].

### Data Collection Statement

The data that support the findings of this study are available from the corresponding author on reasonable request.

## Results

### Patient Characteristics

Data from 161 patients were obtained in total. Successful recanalization (TICI grade 2b–3) was achieved in 139 patients (86.4%).

In patients who were successfully recanalized, the mean (SD) age was 76 (10.9) years, 59% were female, and the mean (SD) baseline NIHSS score was 14.3 (7.5). Eighty-one (58.3%) patients received alteplase prior to EST, and 52 (37.4%) patients received EST only. Further baseline and outcome characteristics of patients with successful reperfusion are displayed in Table 1. Baseline, procedural, and outcome characteristics of the total study population can be found in Supplementary Table 1.

**Table 1:** Demographic, baseline, procedural, and outcome characteristics

	*n* (%)
Demographic characteristics (*n* = 139)	
Age, mean (SD) (years)	76.0 (10.9)
Female sex	82 (59.0)
Premedication	
Antiplatelet therapy	50 (36.0)
Oral anticoagulant therapy	22 (15.8)
Statin	39 (28.1)
Vascular risk factors	
Hypertension	115 (82.7)
Diabetes mellitus	34 (24.5)
Hyperlipidemia	37 (26.6)
Smoking	22 (15.8)
Atrial fibrillation	46 (33.1)
Peripheral artery occlusive disease	14 (10.1)
Premorbid mRS score	
0	47 (33.8)
1	34 (24.5)
2	20 (14.4)
> 2	38 (27.3)
NIHSS score	
Mean (SD)	14.3 (7.5)
Median (IQR)	14.0 (8–20)
Site of arterial occlusion	
ICA occlusion	43 (27.2)
MCA occlusion	95 (68.3)
ICA + MCA occlusion	38 (23.6)
Left side	70 (43.5)
Treatment	
Alteplase only	6 (4.3)
Alteplase + EST	81 (58.3)
EST only	52 (37.4)
Door-to-needle time, mean (SD)^a^ (min)	32.0 (13.0)
Pretreatment ASPECTS^b^	
8–10	89 (65.9)
6–7	31 (23.0)
< 6	15 (11.1)
Median (IQR)	8 (7–10)
Periinterventional aspects	
Conversion to general anesthesia^c^	14 (10.1)
Need of sedation	127 (91.4)
Need of catecholamines	43 (30.9)
MAP reduction of at least 20%^d^	67 (49.3)
MAP reduction of at least 30%^d^	33 (24.3)
MAP reduction of at least 40%^d^	13 (9.6)
MAP < 70 mm Hg	7 (5.0)
Functional outcome parameters (*n* = 139)	
NIHSS score change after 24 h, mean (SD)	− 3.8 (9.0)
mRS score after 3 mo	
0–1	37 (26.6)
0–2	77 (55.4)
0–3	80 (57.6)
In-house mortality	10 (7.2)
Mortality after 3 mo	28 (20.1)
Degree of reperfusion (TICI grade) (*n* = 161^e^)	
0–2a	22 (13.6)
2b	70 (43.5)
2c	19 (11.8)
3	50 (31.1)

ASPECTS, Alberta stroke program early computed tomographic score, EST, endovascular stroke treatment, ICA, internal carotid artery, IQR, interquartile range, MAP, mean arterial pressure, MCA, middle cerebral artery, mRS, modified Rankin Scale, NIHSS, National Institutes of Health Stroke Scale, SD, standard deviation, TICI, thrombolysis in cerebral infarction

^a^Data are missing in 41 patients

^b^ASPECTS were missing in 4 patients

^c^Reasons for conversion are shown in Supplementary Table 2

^d^Data are missing in 3 patients

^e^Data from the whole study population are shown here

Mean SBP, mean DBP, and mean MAP (SD) at baseline were 169.5 (24.5), 92.7 (15.7), and 118.3 (16.5) mm Hg, respectively, which were subsequently lower in each phase of the intervention and post intervention (Table 2 and Supplementary Figure 1). Pre-recanalization MAP reductions of at least 20% occurred in 49.3% of patients (Table 1). The mean BP values of patients who were not sufficiently recanalized are shown in Supplementary Table 3.

**Table 2:** BP variables

Phase	Mean SBP (SD) (mm Hg)	Mean DBP (SD) (mm Hg)	Mean MAP (SD) (mm Hg)	*n*
Baseline	169.5 (24.5)	92.7 (15.7)	118.3 (16.5)	136
Pre-recanalization	155.4 (19.1)	84.1 (11.3)	107.0 (12.5)	139
Post recanalization	151.4 (22.6)	80.6 (13.7)	104.2 (15.0)	136
Post intervention	147.2 (23.3)	76.7 (13.7)	100.2 (15.5)	137

Phase	ΔSBP (SD) (mm Hg)	ΔDBP (SD) (mm Hg)	ΔMAP (SD) (mm Hg)	*n*
Baseline to pre-recanalization	− 13.9 (23.0)	− 8.5 (15.4)	− 10.3 (16.5)	136
Baseline to post recanalization	− 18.0 (26.7)	− 12.0 (16.3)	− 14.0 (18.3)	136
Baseline to post intervention	− 22.3 (29.4)	− 16.0 (17.4)	− 18.1 (19.6)	136

BP data of 3 patients during baseline and 1 patient during post intervention were missing

BP, blood pressure, DBP, diastolic blood pressure, MAP, mean arterial pressure, SBP, systolic blood pressure, SD, standard deviation

### Association of BP with Reperfusion Status

Baseline and pre-recanalization SBP, DBP, and MAP were not associated with the rate of successful reperfusion (Supplementary Table 4).

### Association of BP with Early Neurological Improvement

The mean (SD) change in the NIHSS score after 24 h was − 3.8 (9.0). A higher SBP during baseline (*β* = 0.12 [95% CI 0.04–0.19], *P* = 0.002), pre-recanalization (*β* = 0.18 [95% CI 0.08–0.29], *P* < 0.001), and post recanalization (*β* = 0.12 [95% CI 0.03–0.20], *P* = 0.006), as well as a lower pre-recanalization DBP (*β* =  − 0.19 [95% CI − 0.36 to − 0.02], *P* = 0.03), was significantly associated with less improvement of the NIHSS score at 24 h after admission. Every 10-mm Hg increase in baseline, pre-recanalization, and post-recanalization SBP led to 1.2, 1.8, and 1.2 less improvement in the NIHSS score, respectively.

Higher MAP during baseline (*β* = 0.09 [95% CI 0.00–0.18], *P* = 0.063) showed a trend for association with less improvement in the NIHSS score at 24 h after admission. Mean BP values in other phases showed no association with change in the NIHSS score after 24 h (Table 3 and Supplementary Table 5).

**Table 3:** Association of BP with change of NIHSS score after 24 h

Phase	*β*	95% CI	*P*
SBP during different phases			
Baseline	0.12	0.04 to 0.19	0.002
Pre-recanalization	0.18	0.08 to 0.29	< 0.001
Post-recanalization	0.12	0.03 to 0.20	0.006
Postintervention	0.07	− 0.01 to 0.16	0.103
ΔSBP between different phases			
From baseline to pre-recanalization	0.01	− 0.08 to 0.10	0.759
From baseline to post-recanalization	0.03	− 0.10 to 0.16	0.663
From baseline to postintervention	0.05	− 0.02 to 0.12	0.134
DBP during different phases			
Baseline	− 0.08	− 0.21 to 0.04	0.185
Pre-recanalization	− 0.19	− 0.36 to − 0.02	0.029
Post-recanalization	− 0.12	− 0.26 to 0.02	0.082
Postintervention	− 0.13	− 0.28 to 0.01	0.076
ΔDBP between different phases			
From baseline to pre-recanalization	0.02	− 0.12 to 0.15	0.812
From baseline to post-recanalization	− 0.01	− 0.10 to 0.08	0.845
From baseline to postintervention	0.02	− 0.11 to 0.14	0.781
MAP during different phases			
Baseline	0.09	0.00 to 0.18	0.063
Pre-recanalization	0.08	− 0.04 to 0.21	0.176
Post-recanalization	0.05	− 0.05 to 0.15	0.344
Postintervention	− 0.01	− 0.11to 0.09	0.807
ΔMAP between different phases			
From baseline to pre-recanalization	0.03	− 0.06 to 0.13	0.492
From baseline to post-recanalization	0.04	− 0.05 to 0.12	0.423
From baseline to postintervention	0.09	0.01 to 0.17	0.036

Depicted values are the results of multivariable linear regression analyses, which were performed separately for each phase. Several covariables were used for adjustment and are omitted here for clarity. The full regression models are shown in Supplementary Tables 5 and 6. β depicts the change of NIHSS score after 24 h per mm Hg increase

BP, blood pressure, DBP, diastolic blood pressure, MAP, mean arterial pressure, NIHSS, National Institutes of Health Stroke Scale, SBP, systolic blood pressure

In the analysis of SBP, DBP, and MAP change between the baseline phase and the pre-recanalization, post-recanalization, and postintervention phases, only the degree of decrease in MAP from the baseline to the postintervention phase was associated with less improvement in the NIHSS score (*β* = 0.09 [95% CI 0.01–0.17], *P* = 0.036). With every 10-mm Hg decrease, improvement in the NIHSS score was decreased by 0.9 (Table 3 and Supplementary Table 6).

Reductions of more than 20%, 30%, or 40% in SBP and MAP from baseline during pre-recanalization showed no association with change in the NIHSS score after 24 h in a multivariable regression analysis (Supplementary Table 7).

### Association of BP with mRS Score 0–2 at 3 Months

Seventy-seven (55.4%) patients who received successful reperfusion had a favorable long-term functional outcome, defined as an mRS score of 0–2 after 3 months (Table 1). For every 10-mm Hg increase in baseline and pre-recanalization SBP, there was a lower chance of achieving an mRS score of 0–2 at 3 months, with ORs of 0.76 (95% CI 0.61–0.95, *P* = 0.015) and 0.65 (95% CI 0.47–0.91, *P* = 0.011), respectively. This was also seen for baseline MAP (OR 0.76 [95% CI 0.58–1.00], *P* = 0.05) and pre-recanalization MAP (OR 0.66 [95% CI 0.45–0.96], *P* = 0.03).

Absolute mean BP values in other phases showed no association with the mRS score at 3 months (Table 4 and Supplementary Table 8).

**Table 4:** Association of BP with mRS score 0–2 after 3 mo

Phase	OR	95% confidence interval	*P*
SBP during different phases			
Baseline	0.76	0.61–0.95	0.015
Pre-recanalization	0.65	0.47–0.91	0.011
Post-recanalization	0.84	0.66–1.06	0.136
Postintervention	0.85	0.66–1.08	0.184
ΔSBP between different phases			
From baseline to pre-recanalization	0.95	0.75–1.20	0.642
From baseline to post-recanalization	0.93	0.75–1.16	0.529
From baseline to postintervention	0.88	0.71–1.08	0.222
DBP during different phases			
Baseline	1.12	0.78–1.61	0.539
Pre-recanalization	1.19	0.70–2.00	0.523
Post-recanalization	1.24	0.84–1.82	0.274
Postintervention	1.54	0.99–2.40	0.055
ΔDBP between different phases			
From baseline to pre-recanalization	1.12	0.78–1.61	0.548
From baseline to post-recanalization	0.99	0.69–1.42	0.939
From baseline to postintervention	0.88	0.62–1.25	0.464
MAP during different phases			
Baseline	0.76	0.58–1.00	0.050
Pre-recanalization	0.66	0.45–0.96	0.030
Post-recanalization	0.95	0.71–1.27	0.715
Postintervention	1.10	0.83–1.46	0.499
ΔMAP between different phases			
From baseline to pre-recanalization	1.02	0.78–1.33	0.874
From baseline to post-recanalization	0.90	0.70–1.14	0.376
From baseline to postintervention	0.75	0.58–0.98	0.032

Depicted values are the results of multivariable logistic regression analyses, which were performed separately for each phase. Several covariables were used for adjustment and are omitted here for clarity. The full regression models are shown in Supplementary Tables 8 and 9. OR depicts the change of odds for mRS score 0–2 after 3 mo per 10 mm Hg increase

BP, blood pressure, DBP, diastolic blood pressure, MAP, mean arterial pressure, mRS, modified Rankin Scale, OR, odds ratio, SBP, systolic blood pressure

When the changes in mean SBP, mean MAP, and mean DBP from baseline to pre-recanalization, post recanalization, and post intervention were analyzed, only the decrease in MAP from baseline to post intervention was associated with an mRS score of 0–2 at 3 months. The odds of achieving an mRS score of 0–2 was lower in the group with a larger MAP decrease, with an OR of 0.75 (95% CI 0.58–0.98, *P* = 0.032) for every 10-mm Hg decrease (Table 4 and Supplementary Table 9).

A reduction of more than 20%, 30%, or 40% in SBP or MAP from baseline during pre-recanalization was not associated with the mRS score at 3 months (Supplementary Table 7).

**Fig. 1:**
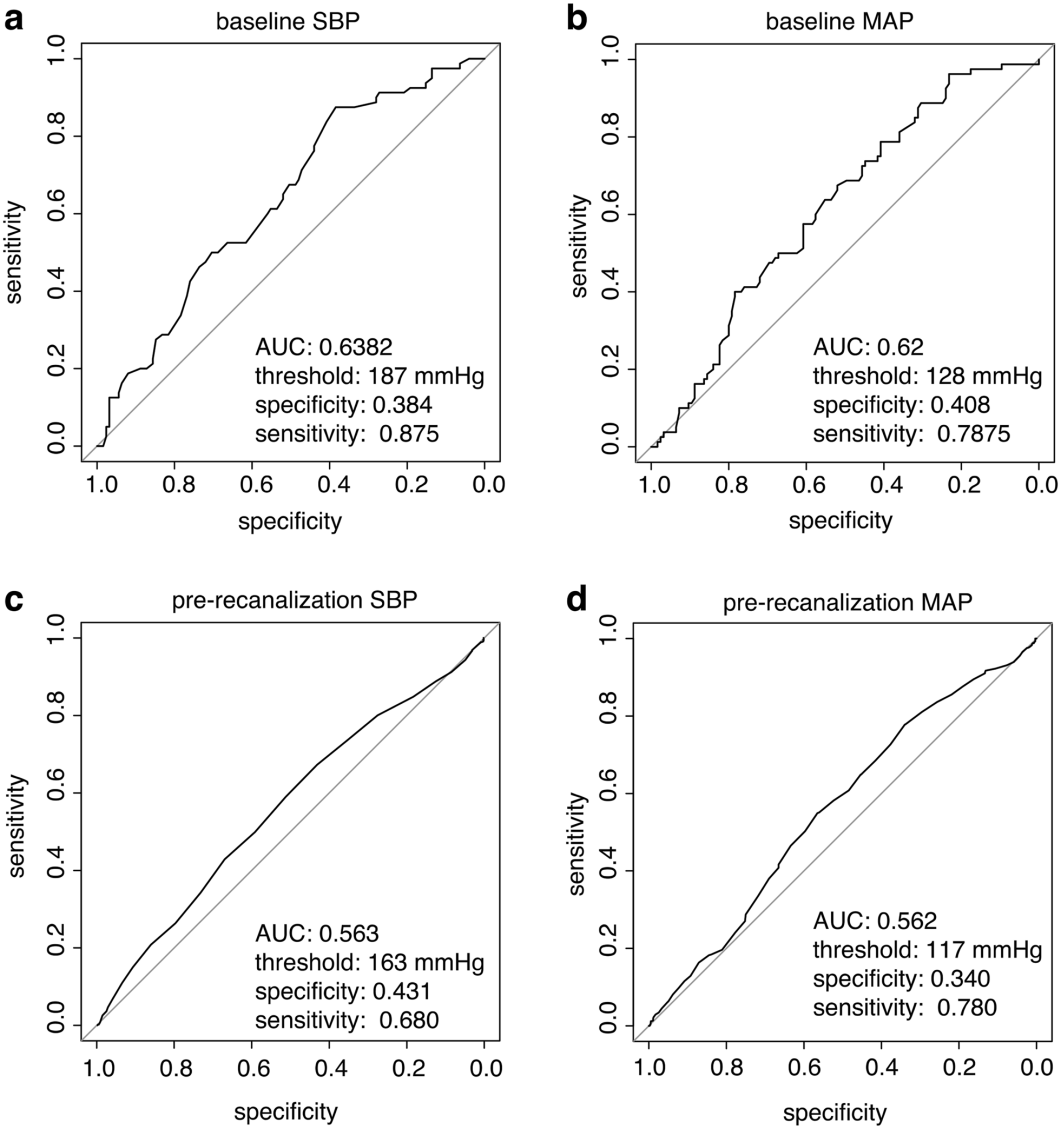
Receiver operating characteristic curves of different blood pressure values at baseline (**a**, **b**) and during pre-recanalization (**c**, **d**) and their sensitivity and specificity to predict a good functional outcome (mRS 0–2) are shown. *AUC* area under the curve, *MAP* mean arterial pressure, *mRS* modified Rankin Scale, *SBP* systolic blood pressure

Threshold values for baseline SBP and MAP discriminating between mRS scores of 0–2 and 3–6 (see Fig. 1) were found to be 187 and 128 mm Hg, respectively, with lower values predicting an mRS score of 0–2. The areas under the curves were 0.64 and 0.62, respectively, and there was a higher sensitivity (0.88 and 0.79) at the expense of low specificity (0.38 and 0.41) for these thresholds (see Fig. 1a, b). For pre-recanalization SBP and MAP, 163 and 117 mm Hg were identified as thresholds, respectively, with lower BP values predicting an mRS score of 0–2 (see Fig. 1c, d). Here, sensitivity (0.68 and 0.78) was also better at the cost of low specificity (0.43 and 0.34), with areas under the curves of 0.563 and 0.562.

## Discussion

The main findings of the study are as follows: (1) Higher SBP during the baseline and pre-recanalization phases was associated with reduced early neurologic recovery and an unfavorable long-term 3-month mRS score. Higher MAP during the baseline and pre-recanalization phases was also associated with an unfavorable 3-month mRS score. Thresholds discriminating favorable and unfavorable functional outcomes were identified as 187 and 125 mm Hg for SBP and MAP on admission, respectively. For the intraprocedural pre-recanalization phase, thresholds of 163 and 117 mm Hg for SBP and MAP, respectively, were found. (2) Reductions or changes in BP from baseline to the pre-recanalization phase of EST showed no association with functional outcome.

### Association of High Pre-recanalization BP with Worse Functional Outcome

An association of higher mean intraprocedural pre-recanalization SBP and MAP with unfavorable functional outcome was found in our study. These findings are not straightforward to interpret, as they are in line with some previous reports but in contrast with another study.

An all stroke U-shaped relationship between BP and outcome has been described for the admission BP, which includes an association of very high BP with a worse outcome [5, 6]. Our statistical approach did not aim at finding a U-shaped relationship, but we also found a worse functional outcome with higher SBP and MAP; this is probably a sign of stroke severity with more unfavorable prognostic factors, such as worse collaterals [18, 19], in which the elevated systemic BP might constitute a compensatory mechanism in maintaining cerebral perfusion.

On the other hand, Pikija et al. [20] showed that higher intraprocedural SBP and MAP (e.g., more than 120% compared with the preprocedure values) led to higher odds of favorable functional outcome. However, their cohort consisted of patients who were treated in general anesthesia, and patients who were unsuccessfully recanalized (TICI grade 0–2b, rate 26.2%) were included in their analysis. A further major difference was that their patients had a lower average SBP on admission (150 vs. 170 mm Hg) and during the procedure (128 vs. 155 mm Hg) compared with our cohort, as well as a lower MAP (107 vs. 118 mm Hg on admission and 91 vs. 107 mm Hg intraprocedurally) [20].

Taken together, BP may affect functional outcome depending on the BP range that the patients were exposed to. If SBP and MAP of patients are situated in a high range, such as in our cohort, higher values might be detrimental for functional outcome, as opposed to patients with BP in lower ranges.

### Intraprocedural Pre-recanalization BP Reductions and Functional Outcome

Most retrospective studies suggested that intraprocedural BP reductions during EST might be associated with worse functional outcome [20–26]. This was mostly interpreted as the importance of maintaining the physiologically elevated BP in the face of large-vessel occlusion to preserve collateral perfusion.

In contrast with these studies, BP reductions were not associated with early or long-term neurological outcome in our study.

Our study cohort differed from those of the other studies in several aspects. Many of the studies mentioned have performed EST nonuniformly under general anesthesia (GA) and mechanical ventilation, which may have led to poorer functional outcome via associations with poor prognostic factors in addition to hypotension (e.g., GA rates of 35% [22] to 100% [21, 24]). However, even patients in whom EST was exclusively performed under procedural sedation, a decrease of 10% or more in MAP from baseline was still associated with poor functional outcome in one study [23]. The latter study had no formal BP protocol, patients were slightly more severely afflicted than in our study (NIHSS score 17 vs. 14, respectively), and average admission BP was situated in a slightly lower range (SBP 158 vs. 170 mm Hg and MAP 107 vs. 118 mm Hg) [23].

Lower admission SBP or MAP (e.g., admission SBP of 140 [24] and 147 [25] vs. 170 mm Hg in our cohort, MAP of 107 [21] vs. 118 mm Hg in our cohort) was also observed in the other retrospective studies, showing a detrimental association between BP reductions and functional outcome. Furthermore, average SBP or MAP during the EST in other studies was also lower than in our study cohort (e.g., procedural SBP of 144 [22] and 119 [24] vs. 155 mm Hg in our cohort and MAP of 75–77 [21] vs. 107 mm Hg in our cohort).

We speculate that in our study, hypotensive BP was counteracted more stringently than in the other studies, and maintaining BP was easier because we mainly treated patients with procedural sedation.

The relationship of BP and BP changes with functional outcome may depend on the exposed BP range. Our findings of associations of worse outcomes with higher intraprocedural SBP, but not with BP reductions, in contrast with the findings of other studies [20–26], possibly support a hypothesis that the type of relationship between BP and functional outcome may depend on which BP range patients are exposed to. Because the BP values of our patients were set in a rather high range, they might represent a subgroup of patients situated in the right ascending part of a possible U-shaped BP–functional outcome relationship, which was described for the admission BP [5, 6]. In a recent meta-analysis of BP data from three randomized clinical trials assessing the anesthetic strategy during EST [SAGA trial team: SIESTA, Anesthesia During Stroke (ANSTROKE), and General or Local Anesthesia in Intra Arterial Therapy (GOLIATH)] [27], prolonged intraprocedural high or low BP was associated with poor functional outcome, further supporting a U-shaped nonlinear relationship of BP and outcome during the intraprocedural phase of EST.

Our findings are especially relevant in the setting of procedural sedation or monitored anesthetic care, in which hypotension is less of a problem than with GA regimens. In our study, we found thresholds of SBP and MAP of 163 and 117 mm Hg, respectively, during the pre-recanalization phase to predict functional outcome, with higher values predicting poor functional outcome. It might be prudent that neurointensivists and neuroanesthesiologists not only pay attention for hypotensive drops but also avoid extremely high BP in patients during EST. However, actual thresholds for harm have to be evaluated in randomized controlled trials, and further studies are needed to dissect the nonlinear relationship between BP and outcome.

On a further note, pursuing absolute BP targets without considering the differences between patients with strokes may be a suboptimal approach, and research on the best individual BP is necessary. It would be interesting for future investigations to study whether each patient has their own BP–outcome relationship during EST with an individual optimal BP corridor because of interindividual differences in occlusion locations, penumbra sizes, collateral status, and other patient-specific characteristics. A randomized trial to investigate an individual approach on optimal intraprocedural BP management has begun recruiting patients (ClinicalTrials.gov identifier NCT04578288) [28].

Limitations of this study comprise the small sample size, the monocentric and retrospective nature of the investigation, and the amount of statistical analysis performed, which limit the rigor of the evidence and can only allow for generating hypotheses that have to be confirmed in randomized controlled studies. Furthermore, BP values were obtained from noninvasive BP measurements and might deviate from invasive arterial measurements. In the analysis of the postinterventional phase, only the first three measured BP values were analyzed. Lastly, observed effect sizes were very small and are, hence, questionable regarding their clinical relevance.

Strengths of this study are the extensive and detailed BP data for different time points in the periinterventional setting of patients with acute ischemic stroke receiving EST. Furthermore, the association of BP and functional outcome was investigated in a homogenous group of patients, with the primary intention to perform EST with a procedural sedation regimen, thus minimizing the influence of GA as a confounder.

## Conclusions

This exploratory study finds detrimental associations between high SBP and MAP during the pre-recanalization phase and functional outcomes after endovascular therapy. The threshold for harm needs further examination in future randomized controlled trials.

## Supplementary Information

Below is the link to the electronic supplementary material.

Supplementary file1 (PDF 390 kb)
