# Supplementary material for: Hemodynamic Status During Endovascular Stroke Treatment: Association of Blood Pressure with Functional Outcome
Source: Neurocrit Care. 2021 Jun 17;35(3):825–34. doi: 10.1007/s12028-021-01229-w (PMC8692300; doi:10.1007/s12028-021-01229-w)
Supplement: Supplementary file 1 — Supplementary file1 (PDF 390 kb) [file 12028_2021_1229_MOESM1_ESM.pdf]

Supplementary material

Table I. Baseline, procedural and outcome characteristics of KEEP SIMPLEST total cohort

Demographic characteristics (n= 161)	
Age, mean (SD), y	75.9 (11.3)
Female sex - no. (%)	96 (59.6)
Premedication - no. (%)	
Antiplatelet therapy	58 (36.0)
Oral anticoagulant therapy	27 (16.8)
Statin	33 (27.3)
Vascular risk factors - no. (%)	
Hypertension	131 (81.4)
Diabetes mellitus	37 (23.0)
Hyperlipidemia	46 (28.6)
Smoking	24 (14.9)
Atrial fibrillation	54 (33.5)
Peripheral artery occlusive disease	14 (8.7)
Premorbid mRS - no. (%)	
0	50 (31.1)
1	41 (25.5)
2	23 (14.3)
>2	47 (29.2)
NIHSS	
Mean (SD)	14.2 (7.6)
Median (IQR)	14 (12)
Site of arterial occlusion - no. (%)	
ICA occlusion	51 (31.1)
MCA occlusion	114 (70.8)
ICA + MCA occlusion	43 (44.1)
Left side	82 (50.1)
Treatment - no. (%)	
Alteplase only	8 (5.0)
Alteplase + EST	90 (55.9)
EST only	63 (39.1)
Door-to-needle time in minutes - mean (SD) *	31.8 (13.0)
Pretreatment ASPECTS – no. (%)<br>n = 160 (ASPECTS was missing in 1 patient)	
8-10	108 (67.5)
6-7	35 (21.9)
<6	17 (10.6)
Median (IQR)	7 (3)
Peri-interventional aspects - no. (%)	
Conversion to general anesthesia	20 (12.4)
Need of sedation	148 (91.2)
Need of catecholamines	53 (30.9)
MAP < 70 mmHg	10 (6.2)
Mean infusion dose/rate of (analgo)sedative medication and vasopressors	
Propofol (n=145)	2.0 mg/min
Esketamine (n=133)	1.0 mg/min
Norepinephrine (n=49)	215.8 ug/min
Remifentanil (n=16)	205 ug/min

*Data was missing in 49 patients

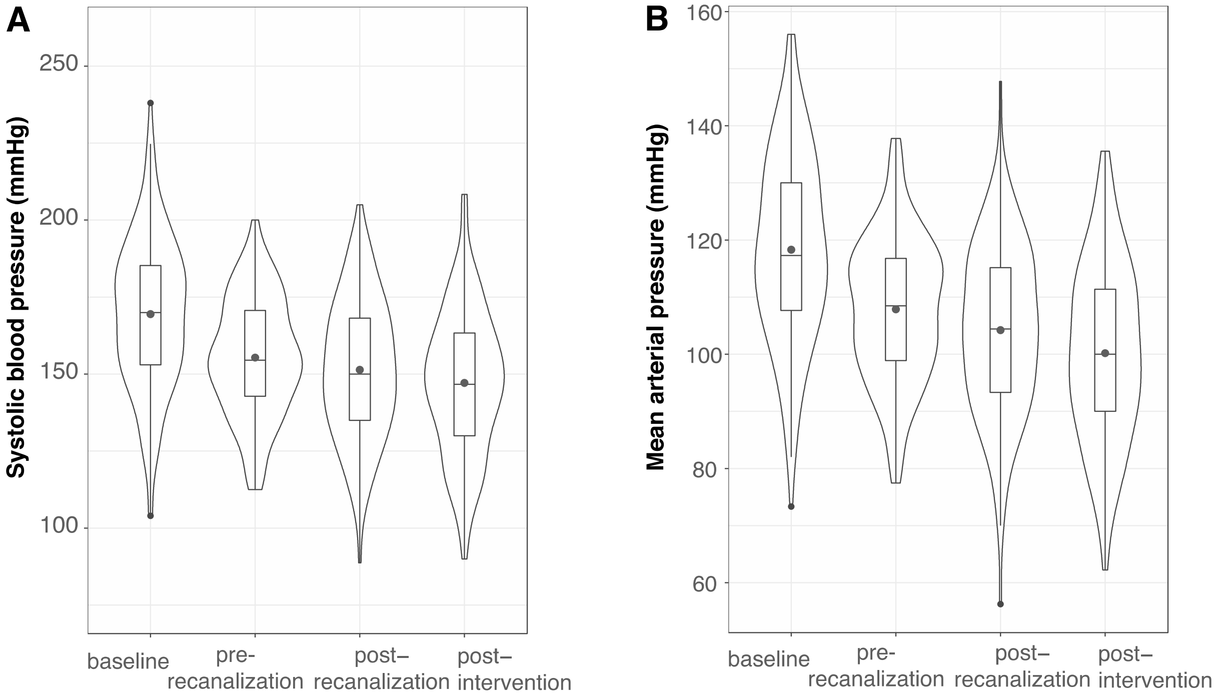

Figure I. Violin plots of systolic blood pressure (A) and mean arterial pressure (B) during different phases of endovascular treatment.

Table II. Reasons for conversion to general anesthesia

Reason, n (%)	Whole study population	Successfully reperfused subgroup
Agitation	11 (55.0)	9 (64.3)
Vomiting	5 (25.0)	3 (21.4)
Aspiration	2 (10.0)	1 (7.1)
Respiratory insufficiency	7 (35.0)	6 (42.9)
other	1 (5.0)*	0 (0)

* one patient was converted to general anesthesia for intracranial stent placement

Table III. Blood pressure in each phase in patients without successful reperfusion (TICI ≤ 2a)

Phase	Mean SBP (SD) in mmHg	Mean DBP (SD) in mmHg	Mean MAP (SD) in mmHg	n
Baseline	175.6 (29.2)	94.4 (19.8)	121.1 (20.8)	22
Pre-recanalization	157.7 (25.4)	86.2 (13.1)	110.0 (15.5)	
Post-recanalization	148.7 (22.1)	82.8 (12.8)	104.8 (14.1)	
Post-intervention	151.6 (17.4)	80.6 (13.1)	104.2 (13.1)	

DBP, diastolic blood pressure; MAP, mean arterial pressure; SBP, systolic blood pressure

Table IV. Baseline and pre-recanalization blood pressure and its association with successful recanalization (TICI 2b-3)

	OR	lower 95-CI limit	upper 95-CI limit	p-value
MAP as covariable during baseline				
MAP	0.841	0.486	1.438	0.528
HR	1.033	0.647	1.709	0.894
Propofol	0.572	0.320	1.012	0.052
Norepinephrine	2.001	0.506	9.965	0.354
Esketamine	0.787	0.433	1.394	0.415
NIHSS baseline	1.133	0.643	2.043	0.669
ASPECTS	0.648	0.354	1.109	0.131
General anesthesia	0.289	0.051	1.506	0.143
SBP und DBP as covariables during baseline				
SBP	0.768	0.409	1.433	0.406
DBP	1.093	0.536	2.269	0.808
HR	0.966	0.574	1.663	0.897
Propofol	0.563	0.313	1.002	0.048
Norepinephrine	1.940	0.492	9.638	0.375
Esketamine	0.810	0.442	1.454	0.483
NIHSS baseline	1.143	0.648	2.067	0.648
ASPECTS	0.656	0.356	1.133	0.148
General anesthesia	0.259	0.043	1.399	0.120
MAP as covariable during pre-recanalization				
MAP	0.630	0.357	1.075	0.097
HR	1.077	0.621	1.957	0.797
Propofol	0.571	0.323	1.007	0.049
Norepinephrine	1.952	0.467	10.419	0.391
Esketamine	0.803	0.441	1.404	0.450
NIHSS baseline	1.072	0.618	1.919	0.808
ASPECTS	0.639	0.344	1.109	0.129
General anesthesia	0.223	0.036	1.210	0.088
SBP und DBP as covariable during pre-recanalization				
SBP	0.651	0.316	1.322	0.236
DBP	0.919	0.439	1.899	0.819
HR	1.010	0.551	1.904	0.975
Propofol	0.551	0.304	0.985	0.043
Norepinephrine	1.870	0.440	10.055	0.425
Esketamine	0.807	0.442	1.414	0.462
NIHSS baseline	1.082	0.623	1.933	0.783
ASPECTS	0.645	0.345	1.126	0.142
General anesthesia	9.755	5.082	21.260	0.078

ASPECTS, Alberta stroke program early CT score; DBP, diastolic blood pressure; HR, heart rate; MAP, mean arterial pressure; NIHSS, national institutes of health stroke scale; SBP, systolic blood pressure

Table V. Blood pressure and its association with NIHSS change after 24 hours

	Estimates	95% CI	p-value
Baseline phase			
SBP and DBP as covariables			
SBP	0.12	[0.04; 0.19]	0.002
DBP	-0.08	[-0.21; 0.04]	0.185
HR	0.03	[-0.05; 0.11]	0.511
NIHSS baseline	-0.37	[-0.57; -0.16]	0.001
ASPECTS	-0.72	[-1.63; 0.20]	0.124
General anesthesia	6.03	[1.21; 10.84]	0.015
MAP as covariable			
MAP	0.09	[0.00; 0.18]	0.063
HR	-0.02	[-0.09; 0.06]	0.666
NIHSS baseline	-0.37	[-0.58; -0.15]	0.001
ASPECTS	-0.58	[-1.51; 0.36]	0.223
General anesthesia	5.06	[0.22; 9.91]	0.041
Pre-recanalization phase			
SBP and DBP as covariables			
SBP	0.18	[0.08; 0.29]	<0.001
DBP	-0.19	[-0.36; -0.02]	0.029
HR	0.04	[-0.06; 0.13]	0.453
Propofol	0.00	[-0.01; 0.02]	0.568
Norepinephrine	-0.30	[-4.16; 3.57]	0.881
Esketamine	0.03	[0.00; 0.05]	0.027
NIHSS baseline	-0.35	[-0.55; -0.15]	0.001
ASPECTS	-0.81	[-1.69; 0.07]	0.073
General anesthesia	3.70	[-1.84; 9.24]	0.191
MAP as covariable			
MAP	0.08	[-0.04; 0.21]	0.176
HR	-0.02	[-0.11; 0.07]	0.663
Propofol	0.00	[-0.01; 0.01]	0.777
Norepinephrine	-1.24	[-5.20; 2.72]	0.540
Esketamine	0.03	[0.00; 0.05]	0.026
NIHSS baseline	-0.36	[-0.57; -0.15]	0.001
ASPECTS	-0.74	[-1.66; 0.17]	0.111
General anesthesia	2.95	[-2.77; 8.67]	0.313
Post-recanalization phase			
SBP and DBP as covariables			
SBP	0.12	[0.03; 0.20]	0.006
DBP	-0.12	[-0.26; 0.02]	0.082
HR	0.01	[-0.08; 0.10]	0.839
Propofol	0.00	[-0.01; 0.02]	0.526
Norepinephrine	-1.28	[-5.15; 2.58]	0.515
Esketamine	0.03	[0.01; 0.06]	0.017
NIHSS baseline	-0.36	[-0.56; -0.15]	0.001
ASPECTS	-0.84	[-1.74; 0.07]	0.069
General anesthesia	3.04	[-2.63; 8.70]	0.294
MAP as covariable			
MAP	0.05	[-0.05; 0.15]	0.344
HR	-0.02	[-0.11; 0.07]	0.675
Propofol	0.00	[-0.01; 0.02]	0.756
Norepinephrine	-1.55	[-5.49; 2.39]	0.441
Esketamine	0.03	[0.00; 0.05]	0.022
NIHSS baseline	-0.36	[-0.57; -0.15]	0.001
ASPECTS	-0.71	[-1.63; 0.21]	0.129

General anesthesia	2.73	[-3.06; 8.51]	0.355
Post-intervention phase			
SBP and DBP as covariables			
SBP	0.07	[-0.01; 0.16]	0.103
DBP	-0.13	[-0.28; 0.01]	0.076
HR	0.02	[-0.06; 0.11]	0.577
Propofol	0.00	[-0.01; 0.02]	0.682
Norepinephrine	-1.51	[-5.35; 2.33]	0.442
Esketamine	0.03	[0.01; 0.06]	0.011
NIHSS baseline	-0.39	[-0.60; -0.19]	<0.001
ASPECTS	-0.81	[-1.70; 0.09]	0.077
General anesthesia	2.43	[-3.48; 8.33]	0.421
MAP as covariable			
MAP	-0.01	[-0.11; 0.09]	0.807
HR	0.00	[-0.08; 0.09]	0.924
Propofol	0.00	[-0.01; 0.01]	0.813
Norepinephrine	-1.36	[-5.24; 2.51]	0.490
Esketamine	0.03	[0.01; 0.06]	0.015
NIHSS baseline	-0.40	[-0.61; -0.20]	<0.001
ASPECTS	-0.87	[-1.78; 0.03]	0.058
General anesthesia	2.50	[-3.47; 8.46]	0.412

ASPECTS, Alberta stroke program early CT score; DBP, diastolic blood pressure; HR, heart rate; MAP, mean arterial pressure; NIHSS, national institutes of health stroke scale; SBP, systolic blood pressure

Table VI. Change of blood pressure between different phases and its association with NIHSS change after 24 hours

	Estimates	95% CI	p-value
From baseline to pre-recanalization			
ΔSBP and ΔDBP as covariables			
ΔSBP	0.01	[-0.08; 0.10]	0.759
ΔDBP	0.02	[-0.12; 0.15]	0.812
HR	-0.01	[-0.12; 0.09]	0.830
Propofol	0.00	[-0.01; 0.01]	0.904
Norepinephrine	-1.71	[-5.69; 2.27]	0.397
Esketamine	0.03	[0.01; 0.06]	0.016
NIHSS baseline	-0.38	[-0.59; -0.17]	0.001
ASPECTS	-0.72	[-1.65; 0.21]	0.127
General anesthesia	2.02	[-3.85; 7.88]	0.497
ΔMAP as covariable			
ΔMAP	0.03	[-0.06; 0.13]	0.492
HR	-0.01	[-0.11; 0.10]	0.888
Propofol	0.00	[-0.01; 0.01]	0.899
Norepinephrine	-1.71	[-5.67; 2.25]	0.394
Esketamine	0.03	[0.01; 0.06]	0.015
NIHSS baseline	-0.38	[-0.59; -0.17]	0.001
ASPECTS	-0.72	[-1.64; 0.21]	0.127
General anesthesia	1.99	[-3.83; 7.80]	0.500
From baseline to post-recanalization			
ΔSBP and ΔDBP as covariables			
ΔSBP	0.03	[-0.10; 0.16]	0.663
ΔDBP	-0.01	[-0.10; 0.08]	0.845
HR	0.00	[-0.01; 0.01]	0.955
Propofol	-1.55	[-5.56; 2.45]	0.445
Norepinephrine	0.03	[0.01; 0.06]	0.015
Esketamine	-0.38	[-0.59; -0.17]	0.001
NIHSS baseline	-0.73	[-1.65; 0.20]	0.122
ASPECTS	1.76	[-4.18; 7.71]	0.558
General anesthesia	0.01	[-0.07; 0.09]	0.859
ΔMAP as covariable			
ΔMAP	0.04	[-0.05; 0.12]	0.423
HR	-0.01	[-0.10; 0.09]	0.882
Propofol	0.00	[-0.01; 0.01]	0.964
Norepinephrine	-1.56	[-5.54; 2.43]	0.441
Esketamine	0.03	[0.01; 0.06]	0.014
NIHSS baseline	-0.38	[-0.59; -0.17]	0.001
ASPECTS	-0.72	[-1.64; 0.20]	0.122
General anesthesia	1.82	[-4.02; 7.67]	0.538
From baseline to post-intervention			
ΔSBP and ΔDBP as covariables			
ΔSBP	0.05	[-0.02; 0.12]	0.134
ΔDBP	0.02	[-0.11; 0.14]	0.781
HR	-0.01	[-0.09; 0.08]	0.891
Propofol	-0.00	[-0.01; 0.01]	0.866
Norepinephrine	-1.02	[-5.03; 2.98]	0.614
Esketamine	0.03	[0.01; 0.06]	0.014
NIHSS baseline	-0.77	[-1.71; 0.17]	0.106
ASPECTS	-0.38	[-0.59; -0.18]	<0.001
General anesthesia	1.17	[-5.01; 7.36]	0.708
ΔMAP as covariable			

ΔMAP	0.09	[0.01; 0.17]	0.036
HR	-0.01	[-0.09; 0.08]	0.866
Propofol	-0.00	[-0.01; 0.01]	0.901
Norepinephrine	-1.14	[-5.11; 2.83]	0.571
Esketamine	0.03	[0.01; 0.06]	0.010
NIHSS baseline	-0.73	[-1.65; 0.20]	0.122
ASPECTS	-0.38	[-0.59; -0.17]	<0.001
General anesthesia	0.77	[-5.31; 6.85]	0.803

ASPECTS, Alberta stroke program early CT score; DBP, diastolic blood pressure; HR, heart rate; MAP, mean arterial pressure; NIHSS, national institutes of health stroke scale; SBP, systolic blood pressure

Table VII. Association of blood pressure drops of at least 20%, 30% and 40% during pre-recanalization vs baseline with early neurological and long-term outcome.

	Estimates	lower 95-CI limit	upper 95-CI limit	p-value
Association of >20% intraprocedural blood pressure reduction with NIHSS change at 24 hours				
MAP reduction	-1.807	-6.705	3.090	0.466
SBP reduction	2.404	-2.483	7.291	0.332
HR	0.112	-1.427	1.652	0.885
Propofol	0.573	-1.520	2.666	0.589
Norepinephrine	-2.088	-6.152	1.976	0.311
Esketamine	2.455	0.374	4.535	0.021
NIHSS baseline	-2.899	-4.637	-1.161	0.001
ASPECTS	-1.278	-2.892	0.336	0.120
General anesthesia	2.683	-3.563	8.928	0.397
Association of >30% intraprocedural blood pressure reduction with NIHSS change at 24 hours				
MAP reduction	-1.241	-6.050	3.567	0.610
SBP reduction	2.779	-2.339	7.898	0.285
HR	0.002	-0.073	0.077	0.963
Propofol	0.003	-0.012	0.017	0.717
Norepinephrine	-2.084	-6.140	1.972	0.311
Esketamine	0.032	0.006	0.057	0.017
NIHSS baseline	-0.371	-0.599	-0.144	0.002
ASPECTS	-0.768	-1.723	0.187	0.114
General anesthesia	2.290	-4.004	8.584	0.473
Association of >40% intraprocedural blood pressure reduction with NIHSS change at 24 hours				
MAP reduction	1.148	-5.168	7.465	0.719
SBP reduction	0.366	-6.979	7.711	0.922
HR	0.008	-1.516	1.532	0.992
Propofol	0.520	-1.599	2.639	0.628
Norepinephrine	-2.028	-6.182	2.125	0.336
Esketamine	2.485	0.399	4.571	0.020
NIHSS baseline	-2.723	-4.444	-1.003	0.002
ASPECTS	-1.241	-2.860	0.379	0.132
General anesthesia	2.256	-3.918	8.429	0.471
	OR	lower 95-CI limit	upper 95-CI limit	p-value
Association of >20% intraprocedural blood pressure reduction with mRS 0-2				
MAP reduction	0.522	0.135	1.894	0.328
SBP reduction	2.976	0.805	12.069	0.110
HR	0.976	0.783	1.200	0.824
Propofol	0.686	0.343	1.273	0.257
Norepinephrine	2.210	0.715	7.041	0.170
Esketamine	0.808	0.422	1.529	0.512
NIHSS baseline	0.883	0.821	0.943	0.000
ASPECTS	1.333	1.016	1.795	0.046
General anesthesia	0.522	0.056	3.455	0.522
Association of >30% intraprocedural blood pressure reduction with mRS 0-2				
MAP reduction	2.361	0.638	9.109	0.200
SBP reduction	0.653	0.153	2.586	0.548
HR	0.938	0.750	1.153	0.557
Propofol	0.723	0.361	1.345	0.330
Norepinephrine	2.388	0.776	7.602	0.131
Esketamine	0.790	0.410	1.504	0.473
NIHSS baseline	0.893	0.831	0.953	0.001
ASPECTS	1.351	1.033	1.811	0.034

General anesthesia	0.383	0.039	2.545	0.352
Association of >40% intraprocedural blood pressure reduction with mRS 0-2				
MAP reduction	1.804	0.224	13.140	0.564
SBP reduction	0.648	0.064	5.764	0.697
HR	0.957	0.772	1.170	0.676
Propofol	0.713	0.362	1.319	0.300
Norepinephrine	2.383	0.764	7.606	0.135
Esketamine	0.831	0.438	1.558	0.562
NIHSS baseline	0.890	0.829	0.950	0.001
ASPECTS	1.314	1.012	1.743	0.047
General anesthesia	0.520	0.059	3.236	0.508

ASPECTS, Alberta stroke program early CT score; HR, heart rate; MAP, mean arterial pressure; NIHSS, national institutes of health stroke scale; SBP, Systolic blood pressure

Table VIII. Blood pressure and its association with modified Rankin scale after 3 months

	OR	95% CI	p-value
Baseline phase			
SBP and DBP as covariables			
SBP	0.76	[0.61; 0.95]	0.015
DBP	1.12	[0.78; 1.61]	0.539
HR	0.94	[0.74; 1.18]	0.572
NIHSS baseline	0.41	[0.25; 0.67]	<0.001
ASPECTS	1.31	[1.00; 1.72]	0.050
General anesthesia	0.31	[0.05; 2.06]	0.225
MAP as covariable			
MAP	0.76	[0.58; 1.00]	0.050
HR	1.02	[0.82; 1.25]	0.885
NIHSS baseline	0.41	[0.25; 0.68]	<0.001
ASPECTS	1.29	[0.99; 1.69]	0.060
General anesthesia	0.50	[0.09; 2.80]	0.434
Pre-recanalization phase			
SBP and DBP as covariables			
SBP	0.65	[0.47; 0.91]	0.011
DBP	1.19	[0.70; 2.00]	0.523
HR	0.83	[0.63; 1.10]	0.205
Propofol	0.98	[0.94; 1.02]	0.340
Norepinephrine	1.49	[0.48; 4.63]	0.494
Esketamine	0.87	[0.41; 1.87]	0.728
NIHSS baseline	0.39	[0.23; 0.65]	<0.001
ASPECTS	1.40	[1.05; 1.89]	0.024
General anesthesia	0.16	[0.02; 1.36]	0.093
MAP as covariable			
MAP	0.66	[0.45; 0.96]	0.030
HR	0.94	[0.73; 1.20]	0.608
Propofol	0.98	[0.94; 1.02]	0.429
Norepinephrine	1.74	[0.57; 5.33]	0.335
Esketamine	0.88	[0.41; 1.86]	0.730
NIHSS baseline	0.41	[0.25; 0.67]	<0.001
ASPECTS	1.37	[1.03; 1.81]	0.030
General anesthesia	0.23	[0.03; 1.75]	0.157
Post-recanalization phase			
SBP and DBP as covariables			
SBP	0.84	[0.66; 1.06]	0.136
DBP	1.24	[0.84; 1.82]	0.274
HR	0.97	[0.75; 1.25]	0.792
Propofol	0.99	[0.95; 1.03]	0.498
Norepinephrine	1.87	[0.62; 5.60]	0.264
Esketamine	0.82	[0.40; 1.69]	0.599
NIHSS baseline	0.41	[0.25; 0.67]	<0.001
ASPECTS	1.34	[1.01; 1.77]	0.042
General anesthesia	0.27	[0.04; 1.92]	0.188
MAP as covariable			
MAP	0.95	[0.71; 1.27]	0.715
HR	1.02	[0.80; 1.30]	0.896
Propofol	0.99	[0.95; 1.03]	0.591
Norepinephrine	2.00	[0.67; 5.99]	0.217
Esketamine	0.82	[0.40; 1.69]	0.592
NIHSS baseline	0.42	[0.26; 0.68]	<0.001

ASPECTS	1.29	[0.99; 1.69]	0.059
General anesthesia	0.32	[0.05; 2.15]	0.242
Post-intervention phase			
SBP and DBP as covariables			
SBP	0.85	[0.66; 1.08]	0.184
DBP	1.54	[0.99; 2.40]	0.055
HR	0.85	[0.67; 1.09]	0.203
Propofol	0.99	[0.95; 1.03]	0.466
Norepinephrine	2.50	[0.80; 7.81]	0.113
Esketamine	0.71	[0.35; 1.43]	0.331
NIHSS baseline	0.41	[0.25; 0.67]	0.001
ASPECTS	1.32	[1.00; 1.75]	0.050
General anesthesia	0.45	[0.06; 3.43]	0.440
MAP as covariable			
MAP	1.10	[0.83; 1.46]	0.499
HR	0.91	[0.72; 1.15]	0.423
Propofol	0.99	[0.95; 1.03]	0.659
Norepinephrine	2.13	[0.70; 6.49]	0.183
Esketamine	0.75	[0.37; 1.51]	0.423
NIHSS baseline	0.45	[0.28; 0.72]	0.001
ASPECTS	1.34	[1.03; 1.76]	0.031
General anesthesia	0.45	[0.06; 3.15]	0.420

ASPECTS, Alberta stroke program early CT score; DBP, diastolic blood pressure; HR, heart rate; MAP, mean arterial pressure; NIHSS, national institutes of health stroke scale; SBP, systolic blood pressure

Table IX. Change of blood pressure between different phases and its association with mRS at 3 months

	OR	95% CI	p-value
From baseline to pre-recanalization			
ΔSBP and ΔDBP as covariables			
ΔSBP	0.95	[0.75; 1.20]	0.642
ΔDBP	1.12	[0.78; 1.61]	0.548
HR	0.98	[0.76; 1.27]	0.891
NIHSS baseline	0.44	[0.28; 0.71]	0.001
ASPECTS	1.27	[0.98; 1.64]	0.066
General anesthesia	0.39	[0.07; 2.07]	0.267
ΔMAP as covariable			
ΔMAP	1.02	[0.78; 1.33]	0.874
HR	0.96	[0.74; 1.25]	0.785
NIHSS baseline	0.44	[0.28; 0.70]	0.001
ASPECTS	1.26	[0.97; 1.63]	0.080
General anesthesia	0.40	[0.08; 2.08]	0.272
From baseline to post-recanalization			
ΔSBP and ΔDBP as covariables			
ΔSBP	0.93	[0.75; 1.16]	0.529
ΔDBP	0.99	[0.69; 1.42]	0.939
HR	1.07	[0.84; 1.35]	0.588
NIHSS baseline	0.45	[0.28; 0.71]	0.001
ASPECTS	1.25	[0.97; 1.62]	0.080
General anesthesia	0.52	[0.09; 2.85]	0.445
ΔMAP as covariable			
ΔMAP	0.90	[0.70; 1.14]	0.376
HR	1.06	[0.83; 1.34]	0.659
NIHSS baseline	0.44	[0.28; 0.70]	0.001
ASPECTS	1.25	[0.97; 1.61]	0.090
General anesthesia	0.52	[0.10; 2.79]	0.439
From baseline to post-intervention			
ΔSBP and ΔDBP as covariables			
ΔSBP	0.88	[0.71; 1.08]	0.222
ΔDBP	0.88	[0.62; 1.25]	0.464
HR	0.97	[0.77; 1.21]	0.757
NIHSS baseline	0.42	[0.26; 0.69]	0.001
ASPECTS	1.30	[1.00; 1.70]	0.052
General anesthesia	0.60	[0.09; 3.84]	0.592
ΔMAP as covariable			
ΔMAP	0.75	[0.58; 0.98]	0.032
HR	0.96	[0.77; 1.20]	0.727
NIHSS baseline	0.42	[0.26; 0.69]	0.001
ASPECTS	1.30	[0.99; 1.70]	0.055
General anesthesia	0.65	[0.11; 3.91]	0.642

ASPECTS, Alberta stroke program early CT score; DBP, diastolic blood pressure; HR, heart rate; MAP, mean arterial pressure; NIHSS, national institutes of health stroke scale; SBP, systolic blood pressure
